# The Evolving Story in the Genetic Analysis for Heart Failure

**DOI:** 10.3389/fcvm.2021.646816

**Published:** 2021-04-13

**Authors:** Kazuo Miyazawa, Kaoru Ito

**Affiliations:** Laboratory for Cardiovascular Genomics and Informatics, RIKEN Center for Integrative Medical Sciences, Yokohama, Japan

**Keywords:** heart failure, cardiomyopathy, genetic architecture, genome-wide association study, big data

## Abstract

Genomic studies of cardiovascular diseases have achieved great success, not only in Mendelian genetic diseases such as hereditary arrhythmias and cardiomyopathies, but also in common diseases such as ischemic heart disease and atrial fibrillation. However, only limited success has been achieved in heart failure due to the complexity of its disease background. In this paper, we will review the genetic research for heart failure to date and discuss how we can discover new aspects of heart failure from the viewpoint of genomic perspective.

## Introduction

In recent years, human genomic research has made great strides, riding the wave of big data. In the field of cardiology, the analyses of hereditary arrhythmias and cardiomyopathies have been very active, and many disease-related genes and causative gene mutations have been identified to date. In particular, cardiomyopathy research could be useful in identifying common mechanisms that cause abnormalities in cardiac function, since the main locus of the abnormality is the myocardium itself (1). But whether it can help us to understand the “common” heart failure (HF) encountered in our daily clinical practice is controversial. On the other hand, with the advent of genome-wide association studies (GWAS), >200 disease-susceptibility loci have been identified thus far in common cardiovascular diseases such as atrial fibrillation (AF) (2) and ischemic heart disease (IHD) (3), which are caused by the accumulation of weak-effect genetic polymorphisms that differ from monogenic diseases such as hereditary cardiomyopathies (Mendelian diseases). Although these advances in technology and methods are gradually revealing the genetic background of many cardiovascular diseases, not many studies have been able to approach the genetic background of “common” HF. This article will briefly review the genetic studies for HF and then suggest how those genetic studies should be viewed in light of the commonality of genetic background between cardiomyopathy and HF.

## Recent Advances in Genomic Research and Cardiovascular Disease

Since the Human Genome Project was completed in 2003 and the outline of the human genome sequence was determined, the researches in this field have developed at an extraordinary pace. In addition to the completion of the reference map of the human genome, the involvement of technologies such as genotyping arrays and next-generation sequencers that can determine genetic variants inexpensively and accurately has made this possible. At the same time, data processing environments, such as computer and data storage, have been improved in the last few years to cope with the growing volume of genomic data. In recent years, large databases of genetic variants including very rare ones have been developed, such as gnomAD (https://gnomad.broadinstitute.org/) (4). Also databases including the information of neighboring omics layers, such as epigenome and transcriptome data that are important to understand genomic information, are now available; ENCODE (https://www.encodeproject.org/), Roadmap Epigenetics (http://www.roadmapepigenomics.org/), GTEx (https://www.gtexportal.org/home/), and other international projects have enriched the data for each organ and cell. Such multi-omics data allow us to infer the function of identified genetic variants without additional wet experiments.

Cardiovascular genomics has also made great strides along these lines. The implementation of targeted resequencing, as well as whole-exome sequencing s and whole-genome sequencing, has dramatically increased the detection rate of mutations that cause hereditary arrhythmias and cardiomyopathies. However, new problems, such as “Variant of Unknown Significance,” have arisen due to the availability of a large amount of genomic data. This problem also seems to be gradually being solved with the advent of organ-specific RNA sequencing (5) and superior *in-silico* prediction algorithms (6, 7). On the other hand, “common” cardiovascular diseases such as IHD and AF are being addressed by large international consortia and mega-biobanks such as the UK Biobank, which have made samples of hundreds of thousands of people available for analysis. The resulting dramatic increase in the statistical power of studies has enabled the identification of many disease-susceptible gene regions (2, 3) and accelerated our understanding of disease mechanisms. And now, there is a growing momentum for clinical applications of genomic information, such as studies using polygenic risk scores, which are genetic risk scores that combine the information of a large number of genetic variants in order to aim achieving precision medicine (8).

## Genomic Research for HF Under Difficult Conditions

While human genomic research is making remarkable progress, genomic research on so-called “HF” seems to have had only limited success. The reason for this is that HF is not a single disease concept but a syndrome that can be caused by a variety of factors, including IHD, valvular heart disease, infectious diseases, congenital heart diseases, and hereditary cardiomyopathies, all of which share a common endpoint. In other words, although the final pathology may be the same, the processes leading to it are different, which makes them incompatible with genomic research that defines the starting point of the disease. Under these circumstances, several HF studies have been conducted, but the full picture of the genetic makeup of HF remains unclear.

Since HF is the end result of many different diseases, it seems like a good idea to consider each disease separately and conduct each genomic research. The less variability there is in the disease, the more statistically advantageous it is and the easier it is to reach a conclusion. However, there is an attempt to identify a common pathway for HF arriving from such a variety of causes. This type of HF is called “all-cause HF (HF),” meaning that no specific cause is assumed. There is also another definition “non-ischemic HF” that excludes only HF caused by IHD. Although the analysis of these disease concepts is inefficient because of the variability in causes, it is expected to discover common pathways for HF independent of the cause. Such studies may lead to the discovery of mechanisms to distinguish between cases that progress to HF and those that do not, in the presence of various types of cardiac stressors.

## What All-Cause HF or Non-Ischemic HF Genomic Studies Suggest

Let's take a look at the status of genomic studies on all-cause HF and non-ischemic HF: among the major GWAS studies (9, 10) conducted until the end of 2018, only three disease susceptibility regions have achieved genome-wide significance levels (*P* < 5 × 10^−8^; Table 1). This is a very small number compared to other “common” cardiovascular diseases such as IHD and AF. However, a careful reading of these can shed light on the nature of HF. The first one, reported by Smith et al. (9), is the disease susceptibility locus (lead variant: rs10519210) located in the intergenic region between *CA12* and *USP3*. Such disease-susceptibility regions identified by GWAS are often not in the protein-coding regions of genes, but in enhancer regions that regulate a gene expression (which may be remote from the target gene). Since an enhancer and the target gene are in close proximity in three dimensions, Hi-C database (11) can indicate the physical proximity to the enhancer region. According to the Hi-C data, the enhancer may act on both *RAB8B* and *FBXL22* genes. Based on the previously reported GWAS (https://www.ebi.ac.uk/gwas/) and the UK Biobank GWAS catalog (https://www.nealelab.is/uk-biobank), *RAB8B* affects AF, systolic blood pressure (SBP), heart rate (HR), and body mass index (BMI), while *FBXL22* affects AF, HR, and BMI. Next, HF susceptibility locus (lead variant: rs1906609) reported by Li et al. was located upstream of the *PITX2* gene, one of the most famous AF-related genes (12), suggesting a genetic relationship between HF and AF. In other words, these two regions reconfirmed clinically known HF risk factors such as AF, SBP, HR, and obesity.

**Table 1:** Genetic loci identified by GWAS for all-cause or non-ischemic HF.

**Study**	**Year**	**The number of samples**	**Chromosome**	**Lead variant**	**Nearest gene(s)**	***P*-value**	**Suggested Associated Traits**
(9)	2010	Case 2,526	15	rs10519210	*USP3*	1.4 × 10^−8^	AF, SBP, HR, BMI
		Control 20,926					
(10)	2018	Case 7,382	4	rs1906609	*PITX2*	9.1 × 10^−10^	AF
		Control 480,628	10	rs121429633	*BAG3*	2.3 × 10^−9^	DCM
(14)	2020	Case 47,309	1	rs660240	*CELSR2*	3.3 × 10^−10^	LDL-C, IHD
		Control 930,014	4	rs17042102	*PITX2/FAM241A*	5.7 × 10^−20^	AF
			5	rs11745324	*KLHL3*	2.4 × 10^−8^	AF
			6	rs4135240	*CDKN1A*	6.8 × 10^−9^	Reduced LV function
			6	rs55730499	*LPA*	1.8 × 10^−11^	LDL-C, IHD
			6	rs140570886	*LPA*	7.7 × 10^−11^	LDL-C, IHD
			9	rs1556516	9p21*/CDKN2B-AS1*	1.6 × 10^−15^	IHD
			9	rs600038	*ABO/SURF1*	3.7 × 10^−9^	LDL-C, IHD, T2D
			10	rs4746140	*SYNPO2L/AGAP5*	1.1 × 10^−9^	AF
			10	rs17617337	*BAG3*	3.7 × 10^−9^	DCM, reduced LV function
			12	rs4766578	*ATXN2*	4.9 × 10^−8^	SBP, DBP
			16	rs56094641	*FTO*	1.2 × 10^−8^	BMI, T2D

*AF, atrial fibrillation; SBP, systolic Blood Pressure; HR, heart rate; DCM, dilated cardiomyopathy; LDL-C, low density lipoprotein cholesterol; IHD, ischemic heart disease; LV, left ventricular; T2D, type 2 diabetes mellitus; DBP, diastolic blood pressure; BMI, body mass index*.

Another non-ischemic HF-related locus (lead variant: rs2234962) reported by Aragam et al. (10) was located on the *BAG3* gene, which is a well-known causative gene for idiopathic dilated cardiomyopathy (DCM). *BAG3* plays a variety of roles in maintaining cardiac function, including linking adrenergic receptors to L-type Ca^2+^ channels, providing structural support for sarcomeres, and inhibiting apoptosis (13). In addition, the *BAG3* gene has also been reported to be associated with other diseases such as Takotsubo cardiomyopathy, HIV-associated cardiomyopathy, and virus-associated myocarditis, in which the myocardium develops cardiomyopathy or myocarditis when exposed to stresses or infection. This suggests that HF and these diseases may share some common genetic background, which means that they may share common disease mechanisms by having the same disease-related genes, and that HF genomics can help us to understand these diseases and vice versa.

## Findings from the Largest HF GWAS in the World

Implications from genomic studies of HF have been useful but, as already mentioned, extremely limited. To overcome this situation, the largest HF GWAS study as of 2020 has been conducted, which is a meta-analysis of global HF studies. In the study presented by Shah et al. (14), 47,309 HF patients and 930,014 control samples were analyzed. As a result, they identified 11 novel disease-susceptibility loci (Table 1). These suggested the associations of HF with IHD and AF from the viewpoint of genetics. They also reported loci involved in myocardial development (*MYOZ1, SYNPO2L*) and cellular senescence (*CDKN1*). In particular, Frey et al. (15) have already suggested in a mouse model that *MYOZ1* regulates exercise performance *in vivo* via modulation of calcineurin/NFAT activity and concomitant changes in myofiber type composition. They also utilized the Mendelian randomization method to show that other factors besides IHD, such as AF, hypertension, BMI, hypertension, and triglycerides, are important in the development of severe HF. Even with this unprecedentedly large sample size, the number of new disease susceptibility loci identified was not so many, and new findings were limited, again highlighting the difficulties of genomic research for HF.

## Measurements of Cardiac Function, Cardiac Morphology, Biomarker for HF, and Genetic Factors

The genetic analysis of all-cause HF was very challenging. On the other hand, genetic analysis using individual cardiac parameters as an outcome variable not only gives more statistical power (i.e., dichotomous vs. continuous variables), but also provides various suggestions regarding cardiac function. Newly, Aung et al. (16) in 2019, Pirruccello et al. (17) in 2020 performed GWAS using parameters obtained from cardiac MRI, which are closely related to cardiac function. They identified 57 new susceptibility loci, 45 of which were novel and had not been reported in previous genomic analyses. In addition, familial cardiomyopathy genes such as *BAG3, FLNC, TTN, GATA4, MYH6, MYH7, NKX2-5, PLN, RBM20*, and *RYR2* were found to be significantly enriched in the susceptibility loci, suggesting an association between genetic factors that define cardiac function in healthy individuals and cardiomyopathy-related genes. They also showed that polygenic risk scores (PRS) derived from the cardiac MRI GWAS were significantly correlated with HF and DCM (it should be noted that the PRS derived from the GWAS of left ventricular diameter in systole (LVDs) was the most correlated). They also showed that these genetic loci also influence cardiac function in patients with cardiomyopathy with low penetrance. Collectively, they demonstrated the association of myocardial measurements with cardiomyopathy genes.

Another structure specific to myocardium that can be observed on MRI is the cardiac trabeculae. This is a complex network of muscular strands thought to be a remnant of embryonic development. Meyer et al. (18) quantified the complexity of myocardial trabeculae using a deep learning algorithm from cardiac MRI images and performed a GWAS. They not only identified loci related to hemodynamic phenotypes and regulation of cytoskeletal arborization, but also showed that trabecular morphology was an important determinant of cardiac performance. Furthermore, they performed a Mendelian randomization study and showed a causal relationship between trabecular morphology and cardiovascular diseases. Their findings were very interesting in that they clarified the significance of myocardial trabeculae on cardiac function and cardiovascular diseases, since the significance of which had not been well-understood before.

In addition to measurements from cardiac imaging, the circulating level of cardiac peptides such as BNP and NT-proBNP, is a useful biomarker used in clinical practice to reflect the state of HF. BNP is also known to have a cardioprotective effect, and its levels increase in worsening HF. Musani et al. (19) identified two loci associated with BNP levels. One of them was the region containing the *NPPB* gene encoding BNP (lead variant: rs198389) as expected, while the other was a missense variant located in *KLKB1* locus (rs3733402). The *NPPB* locus was related to SBP, and the *KLKB1* locus to the aldosterone/renin ratio, both of which were associated with left ventricular mass, suggesting the existence of a shared genetic architecture between these traits. This genomic analysis using cardiac measurements and biomarkers, which does not rely on the conventional case-control analysis, is very meaningful because it shows a new path of genomic analysis for HF and suggests an clear association with hereditary cardiomyopathy.

## Genetic Analysis for Diseases Leading to HF

Other conditions that can cause HF include IHD, AF, and valvular heart disease. IHD and AF have been reported to have more than 100 disease susceptibility loci as already described (2, 3), where IHD has been suggested to be strongly associated with lipids, hypertension, diabetes, inflammation, and other atherosclerosis-related traits, while AF has been suggested to have mechanisms strongly related to HF, such as cardiac development and myocardial contractility, apart from electrophysiological traits. As for valvular disease, Chen et al. (20) have recently identified nine disease-susceptibility loci in aortic stenosis, suggesting the involvement of lipids and the immune system. In addition, there are a few reports on mitral valve prolapse syndrome (21, 22) in which mitral valve repair and cardiac development are reported to be involved in the pathogenesis. These reports suggest that HF-related diseases have both their own unique genetic factors and the shared genetic background, which must be useful in understanding the genetic basis of HF.

## Structural Variations, Non-protein-coding Regions, Epigenome, and HF

Although the studies described so far are mainly based on DNA polymorphisms, we should also consider the influence of alterations in non-protein-coding regions through DNA structural variations, and the influence of the epigenomic changes on cardiac function. Haas et al. (23) conducted a genome-wide evaluation of the association between DNA structural variations (SVs) and gene expression in the human heart and detected SVs affecting non-coding RNAs, protein-coding transcripts and regulatory genomic regions (e.g., enhancers, transcription factor binding sites), demonstrating the importance of SVs on gene transcription in HF. Regarding the effect of epigenetic alterations on HF, Meder et al. (24) used arrays to generate genome-wide DNA methylation profiles of patients with DCM and compared them to controls, and identified 59 differentially methylated CpG loci. Of these, about half each were hypomethylated or hypermethylated, indicating a variety of directions of DNA methylation associated with HF. In addition, the authors identified 517 epigenetic loci associated with DCM and cardiac gene expression. This study also reported overlapping methylation patterns between myocardium and peripheral blood, including demethylated *NPPA* and *NPPB* loci, which encodes an important cardiac stress marker ANP and BNP, respectively. Thus, epigenomic information seems to be more dynamic than expected and may be responsible for various pathological conditions in HF.

## *TTN* Gene and HF

The *TTN* gene is one of genes that encode proteins that make up the sarcomere, the contractile unit of the heart muscle. The sarcomere genes such as *MYH7* and *MYBPC3* are known to be causative for hereditary cardiomyopathies. Among them, the *TTN* gene is so large that it was not comprehensively studied in the days when only Sanger sequencing was available. When high-throughput sequencers started to become available, targeted resequencing of the *TTN* gene was performed. Then, it was reported that about 25% of DCM was caused by *TTN* protein truncating variants (25). Subsequently, it was reported that about 15% of DCM was caused by *TTN* gene abnormalities (26). Although the percentage decreased in the later report, it remained the most common of all responsible genes for DCM in both adults and children. However, there was no report of DCM caused by *TTN* mutation in infants, suggesting that *TTN* truncating variants themselves do not immediately cause DCM, but rather have characteristics that develop as a result of a certain amount of stress or after a certain amount of time has passed. It has also been reported that *TTN* truncating variants are relatively common even in healthy individuals, where as much as 1% of them have *TTN* mutations (27). Whether or not *TTN* truncating variants cause cardiomyopathy has been proven, to some extent, to depend on the region of the TTN protein where the mutation is located. The A- or I-band distal ends of titin proteins, which have high exon utilization, have high odds ratios for the disease development due to protein truncation mutations. These effects of TTNtv are thought to be due to “poison peptide” or haploinsufficiency. In addition, the accumulation of the effects of common variants affects the phenotype of TTNtv (17).

According to a report by Schafer et al. (28) in 2017, a comparison of cardiac MRI images of healthy subjects with and without TTNtv revealed that those with TTNtv had a small but significant change in left ventricular morphology. They then showed in rats that such TTNtv can cause cardiac dysfunction under stress. The results also suggest that TTNtv may cause haploinsufficiency due to nonsense-medicated decay regardless of its position (even on the I-band). Collectively, they demonstrated that TTNtv, which are normally considered to be unproblematic, can define the stress response and lead to HF under stress. Ware et al. (29) analyzed perinatal cardiomyopathy, a condition that causes HF in pregnancy, and reported that two-thirds of the causative gene mutations were *TTN* truncating variants. Another report showed that *TTN* truncating mutations were identified in patients with adriamycin cardiomyopathy who developed HF due to the administration of the anticancer drug (30). In perinatal cardiomyopathy and adriamycin cardiomyopathy, patients who originally had no abnormal cardiac function develop HF upon stress loading such as pregnancy or administration of anticancer drugs. In other words, this is consistent with the characteristic of *TTN* mutations, which does not cause any particular problem under steady state conditions, but develops HF when stressed. Thus, the *TTN* mutation may determine the stress response of the myocardium.

Additionally, *TTN* is a well-known splicing target of the protein encoded by *RBM20*, which contains zinc finger domains, an RNA recognition motif (RRM), a serine- and arginine-rich region (RS region), a leucine-rich region, and a glutamate-rich region. These regions, especially the RS region, are highly conserved among species, and mutations in these regions can lead to loss of RBM20 function and the development of DCM (31). It has also been reported that other splicing targets of RBM20 including important DCM-related genes, *CACNA1C* and *CAMK2D*, may be involved in the pathogenesis of cardiomyopathy caused by *RBM20* mutations (32). Specifically, during transcription, TTN pre-mRNA and RBM20 form clusters that spatially attract other RBM20 targets on different chromosomes such as *CACNA1C* and *CAMK2D* loci. Furthermore, this colocalization leads to increased splicing activity of RBM20. Conversely, the loss of TTN pre-mRNA causes splicing changes in *CACNA1C* and *CAMK2D* similar to the loss of RBM20 itself. This mechanism promotes alternative splicing of RBM20-dependent transcripts, suggesting the existence of a cardiac-specific trans-interacting chromatin domain that functions as a “splicing factory.”

## Discussion

**Figure 1:**
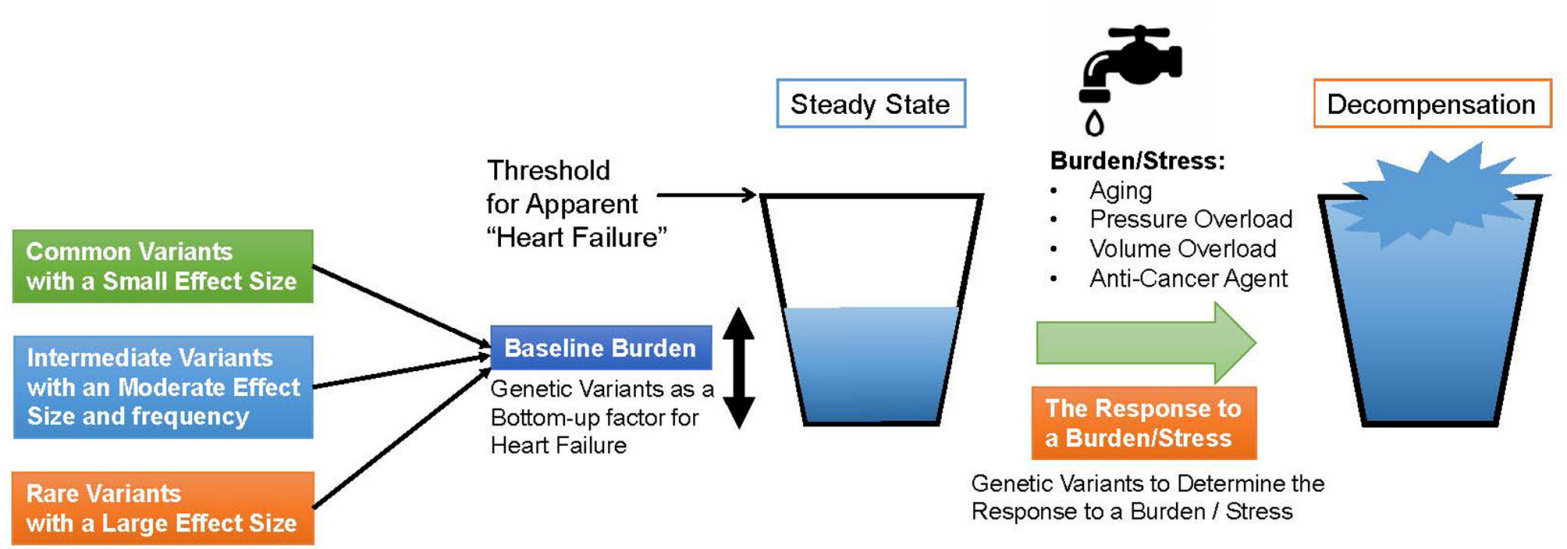
A model for heart failure development. The horizontal line denotes a threshold for apparent heart failure. First, genetic Variants determine the baseline burden of the heart and serve a bottom-up factor for heart failure. If a patient has a cardiomyopathy-causing variant, the baseline burden itself may exceed the threshold. When the heart is subjected to a burden or a stress such as aging, pressure or volume overload and anti-cancer agent, the amount of the burden grows up and exceeds the threshold. In the process, genetic variants determine the response to the burden or the stress.

Genomic studies of HF not only traced known clinical risk factors, but also revealed a common genetic basis with various cardiomyopathies and myocarditis, because of the shared causative genes such as *BAG3* and *TTN*. These similarities lead to the following findings. These commonalities suggest that (i) HF has a common pathway, (ii) some cardiomyopathies, which share some genetic basis with HF, develop under load and may define baseline load and stress response of the heart, and (iii) it may define the baseline morphology and function of the myocardium. From these properties, we should further try to prove the hypothesis that genetic factors, identified in the genomic studies of all-cause HF and non-ischemic HF, play a shared role in the development of various types of HF, as shown in Figure 1. In this model, under steady-state conditions, HF-associated genetic variants act as “bottom-up” factors in HF and define the baseline burden. In the steady state, the threshold for HF is not exceeded, and HF symptoms do not develop. HF-associated genetic variants include common variants [minor allele frequency (MAF) >5%], which have been identified through GWAS and generally have a small effect size but have a high frequency of being associated with disease risk; intermediate effect variants (MAF 1–5%), which have a moderate effect size and frequency between common variants and rare disease variants; rare (MAF <1%) and highly effective variants that cause Mendelian disease. As mentioned earlier (17), factors such as common variants and intermediate effect variants have also been shown to affect the penetrance of individuals with Mendelian disorders by pushing the genetic burden toward the threshold of the disease. When the myocardium is further stressed by aging, pregnancy, pressure overload, volume overload, anticancer drugs, etc., the threshold is exceeded and HF develops. Here, HF-related gene mutations define the stress response. In other words, through genomic studies of HF we can learn about the genetic underpinnings that define the basic characteristics of cardiac function and determine susceptibility to the development of HF.

Thus, genomic studies can provide interesting insights into our understanding of human HF. However, even with the world's largest HF GWAS study, the number of novel disease susceptibility loci was not so many, and the mechanisms of disease susceptibility identified in this study were limited to tracing previous discoveries. Therefore, it is important to promote HF genomic research in a more multifaceted manner by integrating other omics data and using methods such as machine learning in addition to conventional frequency-based statistical methods.

## Author Contributions

KM and KI wrote the manuscript. Both authors contributed to the article and approved the submitted version.

## Conflict of Interest

The authors declare that the research was conducted in the absence of any commercial or financial relationships that could be construed as a potential conflict of interest.
